# Digital taxation to promote frugal innovation in institutions of higher learning: a three-decade systematic literature review

**DOI:** 10.12688/f1000research.73318.1

**Published:** 2021-10-18

**Authors:** Wei Ling Kwan, Magiswary Dorasamy, Abdul Aziz Bin Ahmad, Jayamalathi Jayabalan, Pradeep Kumar, Lingeswaran Subermaniam

**Affiliations:** 1 Faculty of Management, Multimedia University, Cyberjaya, Selangor, 63100, Malaysia; 2 Faculty of Accountancy and Management, Universiti Tunku Abdul Rahman, Kajang, Selangor, 43000, Malaysia; 3 Jai Barath Art and Science College, Perumbavoor, Kerala, 683556, India; 4 L Corporate Boards Sdn Bhd, Nilai, Negeri Sembilan, 71800, Malaysia

**Keywords:** Digital Taxation, Frugal Innovation, Institutions of Higher Learning, Educational Ecosystem, Systematic Literature Review, Industry Revolution 4.0, Digital Transformation, Digitisation

## Abstract

**Background:** In the era of the Fourth Industrial Revolution (IR 4.0), digital taxation emerged as a tool for accelerating the economic growth of a nation. While Industry 4.0 focuses on enabling real-time decision-making with sophisticated technology to enhance productivity, digital taxation can serve as an important tool for improving business sustainability. Institutions of higher learning (IHL), which aim to design an IR 4.0 educational ecosystem, can embrace digital taxation, as they face various challenges with different resources. The literature indicates that frugal innovation through digital taxation in institutions of higher learning, can solve emerging resource challenges.

**Method:** We present a systematic review of studies on digital taxation to promote frugal innovation published in the past three decades (1991 to 2021). We obtained a total of 21 papers from a ‘digital taxation’ keyword search, 10 of which were related to digital taxation. However, the 10 papers were not related to frugal innovation.

**Result:** We present two major findings. Firstly, research on digital taxation for frugal innovation is scant. Secondly, challenges exist in digital taxation implementation, which requires further attention.

**Conclusion:** We conclude this review with a recommendation for the conceptual framework, to highlight potential research warranting the attention of the research community.

## Introduction

The rapid growth of institutions of higher learning (IHL) in Malaysia, as well as the intense competition between them, compelled considerable attention to be paid to the provision of high-quality services. ^1^^,^^2^ IHL are seen as crucial in the development of highly skilled talents and the socio-economic status of a nation. Numerous private IHL are facing critical issues, such as decreasing funding, deteriorating teaching conditions and educational facilities, declining student enrolment rates, dwindling numbers of experienced professors, and increasing unemployment rates among university graduates. ^3^ When faced with a reduction in revenue and high expenses for delivering excellent education, resource constraints and financial challenges, IHL are motivated by profits to sustain themselves. IHL must pay considerable attention to creating value for their stakeholders through frugal innovation and digital taxation in response to the dynamic business environment. Engaging in frugal innovation is crucial for IHL to transform constraints into advantages, by minimising the use of resources through digital taxation.

### Frugal innovation

The Oxford Dictionary defines ‘frugal innovation’ as ‘sparing or economical as regards money or food’. Frugal innovation involves not only reforming products but also reconsidering the entire production process and business model. The philosophy of frugal innovation originated from the perspective of emerging economies and has become a concern for people in developed economies. ^4^ The characteristics of frugal innovation, such as low cost, multiple core functionalities and optimised performance, generated emerging concerns about the idea of frugal innovation in the market. ^5^ Besides, frugal innovation consists of environmental benefits and business opportunities. ^6^ Although fugal innovation lacks high-technology features, it can satisfy people’s basic needs at a low cost with comparably high value. ^7^ Thus, exploring the contribution of frugal innovation to emerging countries is necessary.

An example of a successful frugal innovation idea can be seen in post-Second World War Japan, in the Japanese lean processes. During the time of limited natural resources, constrained international access and restricted space and funds, Japanese firms developed the famous concepts of ‘lean’, ‘just-in-time manufacturing’, ‘miniaturisation’ and ‘ *kaizen’*. ^8^ According to recent innovation studies, frugal innovation is becoming popular in emerging countries, owing to their growth potential and business opportunities. ^9^ The majority of firms in developing countries is small and medium sized, ^10^ which promotes the positive motivation to examine less costly factors influencing organisations’ innovation in such countries. ^11^ Frugal innovation can be achieved through the practice of digital taxation. ^12^

### Digital taxation

Digitalisation can affect tax policies and tax administration at the domestic and global levels. With new challenges from the current environment, digitalisation will have numerous implications for taxation. ^13^ Digitalisation is converting various aspects of everyday life as well as the organisation and functions of the economy and society. In its 2019 national budget, the Malaysian government announced that digital tax would be implemented in January 2020. Singapore, followed by Malaysia, are the only Southeast Asian countries that introduced a tax scheme for the digital sector. Value-added tax (VAT) or goods and services tax is currently the most common consumption tax utilised internationally. Among different worldwide players in the digital sector, digital tax will induce taxation asymmetry. For example, Netflix is taxed, but other companies providing online advertisements or social network operators are not included in digital taxation. In consideration of this situation, before developing a proper indirect international tax system, governments around the world should pay attention to the benefits of digital tax revenue as well as taxation distortions from other aspects. ^14^

Digital tax exemption is allowed for online distance learning (ODL) in IHL. IHL promote high-quality intellectual growth, stimulate high-standard research and produce general knowledge and expert human workforce for different industries. In Malaysia, IHL can be divided into public and private universities. Public universities are fully supported by the federal government and public sector. Currently, Malaysia has 20 public universities with curricula for various disciplines. Meanwhile, numerous private universities contribute to the demand for tertiary education. ^15^

### Challenges of digital taxation in IHL

Digital taxation comprises policies on special tax rates for organisations that offer products and services digitally. To guarantee a neutral tax policy for all businesses, such policies extend current rules, such as when a country extends its VAT to include digital services. In addition, special corporate tax rules plan to recognise digital companies with a permanent establishment, despite lacking a physical presence. With the development of the digital economy, tax policies for digital companies also emerged. For example, tax policies are encouraged for business models such as social media companies, and e-commerce and network-based service platforms. ^16^ For the purpose of this study and analysis, digital tax is categorised as shown in Table 1.

**Table 1.** Types of digital tax. ^16^

Types of digital tax	Description
Consumption taxes	Value-added taxes (VAT) and other taxes on the sale of final goods or services are consumption taxes. The consumption taxes have been growing to include digital goods and services.
Digital services taxes	The gross income taxes with a tax base, which comprise income gain from a particular digital goods and services, or according to the number of digital users in a nation, is known as digital services taxes.
Tax preferences for digital business	Tax preferences, are the policies for example like research and development (R&D credits, which lower the tax burden on digital business.
Digital permanent establishment rules	These are the policies on anything creates a permanent establishment for include digital organization that present virtually within a control.

Digital taxation can promote frugal innovation in IHL through tax exemption policies for ODL. ODL is a method of delivering online education in a flexible manner through a combination of synchronous and asynchronous modes, to enable learners to acquire knowledge regardless of their location. The Malaysian government employs ODL to embrace digital services and encourage online education. The government offers service tax exemption to ODL services for all IHL. This policy substantially influenced IHL to offer low-cost educational services. As working adults also require knowledge enrichment, a high demand for ODL emerged. Technological devices such as smartphones, computers and the Internet serve as the main media for delivering and improving ODL. In other countries, such as Tanzania, governments provide tax exemptions for all imported educational and technological devices. ^17^ Upon fulfilling VAT authorities’ requirements for digital services, IHL need not automatically comply with VAT policies. Thus, VAT exemptions relating to educational services are implemented within VAT jurisdictions. However, foreign jurisdictions typically do not provide such wide tax exemptions, such as in the United States, owing to the narrow definition of exemptions for education. Thus, IHL must pay attention to their delivery modes and target students to determine whether their distance learning programme will be exempted.

Given this background, the research questions of this study were as follows:

1. What are the factors influencing digital taxation for ODL in the IHL educational ecosystem?
2. What are the challenges of digital taxation for frugal innovation in the IHL ecosystem?
3. Does a research gap exist in digital taxation for frugal innovation in the IHL ecosystem?

The objectives of this proposal were as follows:

1. To identify the factors influencing digital taxation for ODL in the IHL educational ecosystem
2. To examine the challenges of digital taxation for frugal innovation in the IHL ecosystem
3. To investigate the research gap in digital taxation for frugal innovation in the IHL ecosystem

We examined a total of 21 articles relating to digital taxation to promote frugal innovation in IHL and recommend two outcomes from the current literature. The first outcome is factors affecting digital taxation for frugal innovation, and the second outcome is challenges of digital taxation for frugal innovation. Research on the role of digital taxation in IHL is limited.

## Methods

This paper was designed to present a literature review, a research gap analysis and the obtained insights, to examine the effects of digital taxation on frugal innovation. Using knowledge-based theory and the dynamic capability theoretical lens, key factors influencing digital taxation for frugal innovation in IHL were explored. The five stages of the systematic review recommended by ^18^ involve the following steps:

• Planning the review
• Identifying and evaluating studies
• Extracting and synthesising data
• Reporting the descriptive findings
• Utilising the findings to inform the research and practice

### Stage 1: planning the review

The main purpose of this review was to determine the nature of the research on digital taxation to promote frugal innovation in IHL. This study aims to provide academics with a complete review of past works linked to digital taxation to promote fugal innovation in IHL, specifically the types of policies established. The outcome of the review process aims to provide digital taxation authorities with a series of research insights to continue their development of the field.

### Stage 2: identifying and evaluating studies

In identifying current studies analysing the role of digital taxation in frugal innovation in IHL, the main concern was that most studies lacked knowledge on the type of tax used for digital products and services. Taxes depend on the role of a business, such as consumption tax, digital services tax, tax preferences for digital businesses, digital permanent establishment rules and gross-based withholding tax on digital services. However, the definition of digital taxation includes all the aforementioned roles. Thus, we examined all articles for the selected keywords, from general (‘digital taxation’) to specific (‘digital taxation for IHL’) keywords.

This review analysed empirical research on digital taxation, specifically digital taxation for IHL.

*Keywords*

This review focused on three main research areas, which are, (1) ‘digital taxation’, (2) ‘frugal innovation’ and (3) ‘university’, To find related articles, in the digital taxation and frugal innovation in IHL structure, focus categories and component title keywords were adopted. Table 2 presents the focus categories.

**Table 2.** Selected keywords.

Focus category			
1	2	3	4
Digital taxation	Frugal innovation	Frugal innovation,	Frugal innovation, digital taxation
		Digital Taxation	University
Digital services	Frugal innovation	Digital services	Digital services
Tax or digital taxation	or	Tax or digital Taxation	Tax or digital taxation
	frugal innovations	and frugal innovation	and frugal innovation in university

*Search strategy*

A search strategy was employed to examine articles on digital taxation for frugal innovation in major online databases. A total of five online databases containing considerable taxation research and studies related to IHL were searched, and the search results are presented in Table 3.

**Table 3.** Summary of keyword search.

No.	Online database	Keywords combinations				Unit of analysis
		Digital taxation	Frugal innovation or FI	Frugal innovation and Digital taxation	Frugal innovation and Digital taxation and University	(Selected papers)
1	Emerald	20	200	0	0	13
2	ProQuest	4	158	0	0	6
3	Scopus	10	384	0	0	1
4	Science Direct	3	316	0	0	3
5	Springer	2	11	0	0	0
	Total:	39	1069	0	0	23

*Inclusion and exclusion criteria*

**Figure 1.**
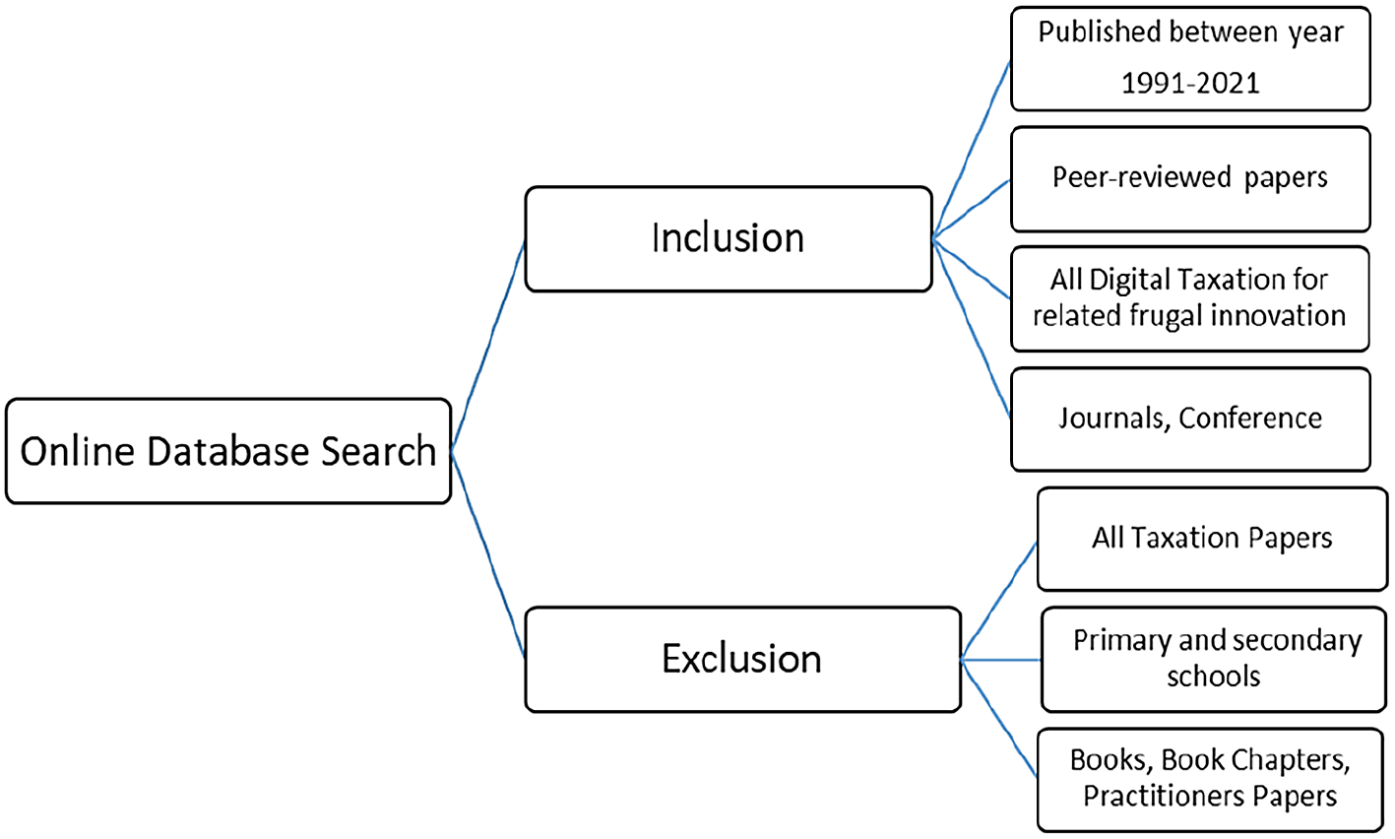
Inclusion and exclusion criteria.

For the purpose of this review, Figure 1 illustrates the inclusion and exclusion criteria for the papers. The papers selected were limited to those published in the past three decades, peer reviewed and on digital taxation related to frugal innovation, and journal and conference papers. Two reviewers screened each of the retrieved articles cooperatively.

### Stage 3. Extracting and synthesising data

**Figure 2.**
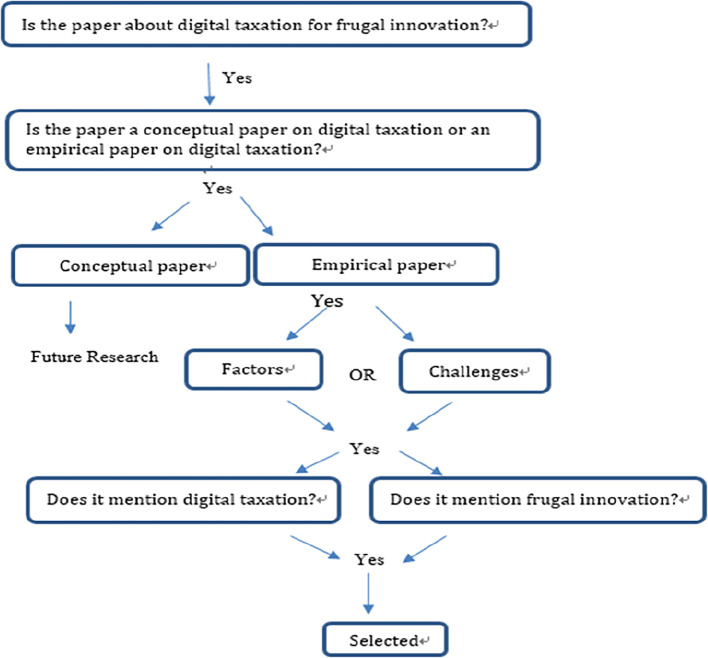
Extraction process.

**Figure 3.**
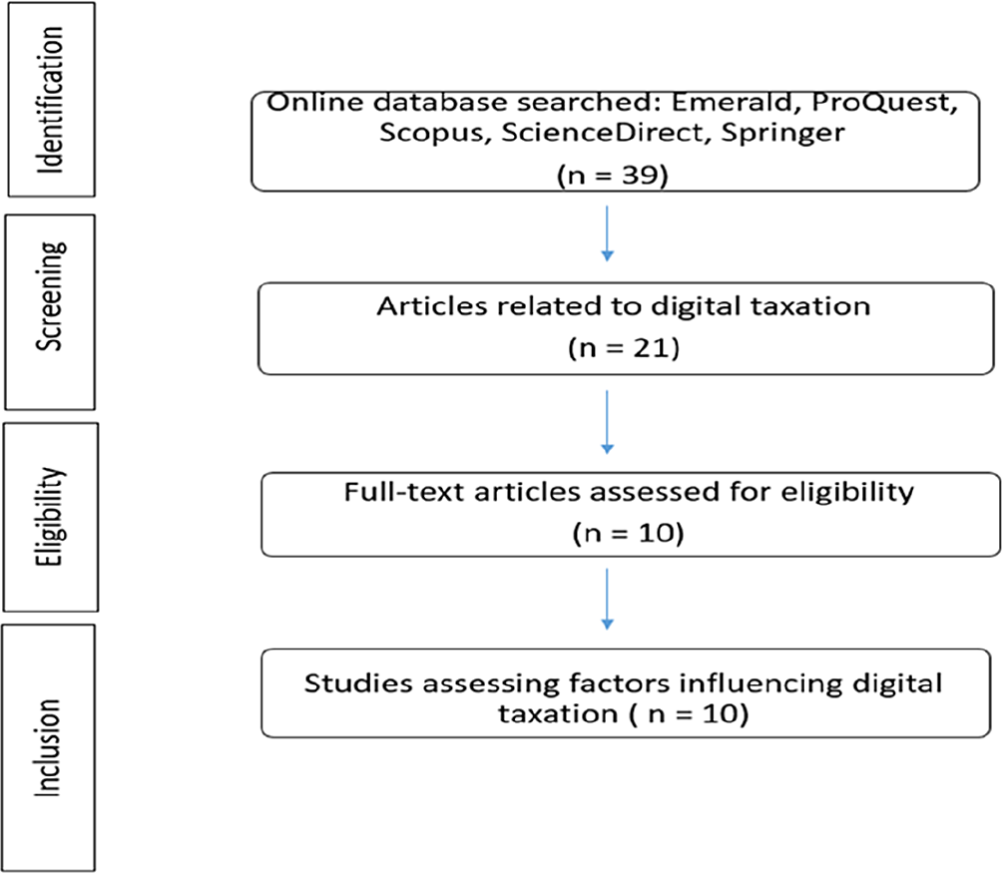
Prisma flow diagram.

The papers from the sources mentioned above were selected based on the aforementioned criteria, as shown in Figure 2. The basis for selecting a paper for review is depicted in Figure 2. From the major databases and other sources, only the digital taxation papers related to frugal innovation/IHL were selected for further review. The following subsection reports the relevant papers chosen according to the selection criteria. Among all the papers obtained, only 10 were retained for the final review. The remaining 11 papers were excluded as there were no relation to FI concept. Stages 4 and 5 of the Transfield process are presented in the following sections ^27^ ( Figure 2). Figure 3 Prisma flow diagram illustrates the flow of paper identification. We categorized the content of the articles by paper sections, such as methods, and by research components, such as factors.

### Ethical considerations

The study was conducted according to the guidelines and approved by the Research Ethical Committee of Multimedia University, Cyberjaya, Malaysia (EA1372021).

## Results

### Finding 1: Factors influencing digital taxation for frugal innovation

According to the inclusion criteria and extraction process stated above, the potential unit for analysis was 10 papers, only four of which described digital taxation factors related to frugal innovation. The factors in the papers were gross domestic product growth, gross savings, VAT, gross state domestic product (GSDP), firm size, connection with the government and gross domestic product. Some of the repeated factors were gross domestic product and VAT. This outcome shows the existence of core factors in the research on digital taxation related to frugal innovation. However, in the examined research papers, the term ‘frugal innovation’ was not explicitly mentioned, but the concept was discussed. The four research papers are listed in Table 4.

**Table 4.** Digital taxation factors related to frugal innovation.

Paper	Factors
^19^Shahid *et al.* (2019)	Gross Domestic Product Growth, Gross Savings
^21^Khan and Shadab (2013)	VAT, Gross State Domestic Product (GSDP)
^25^Hajdúchová *et al.* (2015)	VAT calculation: VAT = price without tax × VAT rate VAT=price with tax/coefficient
^27^Wang *et al.* (2021)	Firm size, Firm age, Connections with government, Gross Domestic Product

### Finding 2: Challenges of digital taxation for frugal innovation in IHL

From the 10 papers selected for review, only one described the challenges of digital taxation. Based on the keyword combination search in Table 3, no papers on digital taxation for frugal innovation were found. Thus, we present only the current research papers on the challenges of digital taxation. This outcome is the same as in Finding 1. We found only one paper on the challenges of digital taxation, with the concept only indirectly related to the term ‘frugal innovation’. The results are shown in Table 5.

**Table 5.** Challenges of digital taxation.

Paper	Challenges
^24^ Beebeejaun (2021)	“Affordability obstacles are faced by customers as digital taxes add on to the cost of ownership of digital services”
	“A reduction in infrastructure investment by foreign suppliers as these taxes decrease the amount available for capital expenditure”
	“Slowing down of the adoption of new technology in countries where digital taxes are imposed, which may hinder economic growth and development of such countries”
	“The emergence of abusive tax avoidance schemes which leads to loss of tax revenue to host countries where digital services are being provided”

### Summary of 10 core papers

An analysis of the 10 papers related to digital taxation for frugal innovation/IHL is presented in this review to identify previous studies related to digital taxation for frugal innovation. The final unit of analysis is 10 papers, and the result are listed in Table 6.

**Table 6.** Summary of core papers.

No	Authors (Year)	Theory	Respondent	Factors/variables	Findings	Method
1	^19^Shahid *et al.* (2019)	Consumption tax and its effects on economic growth rate	Use data from world development indicators, IMF and OECD	Gross Domestic Product Growth,	Consumption tax important to economic	Model based
2	^20^Hodzic *et al.* (2017)	Value-Added Tax and its efficiency	Data from EU-28 Member States and Turkey	-	VAT system need to be reformed	Model based
3	^12^Bhattarai *et al.* (2019)	Impacts of direct and indirect tax reforms	Use data from world development indicators, OECD	-	VAT can achieve budget goal	Model based
4	^21^Khan and Shadab (2013)	Impact of value-added tax (VAT) revenue	Data from government source, Reserve Bank of India report	VAT, Gross State Domestic Product (GSDP)	VAT can improve tax compliance	Model based
5	^22^Kusumanto (1989)	Incidence of value-added tax in indonesia	-	-	VAT bring positive impact	Model based
6	^23^Maria (2020)	Forecast of budget revenue from taxes in digital	-	-	Tax planning have to improved	Design
7	^24^Beebeejaun (2021)	VAT on foreign digital services	Use data from world development indicators, OECD		Imposing of VAT	Case study
8	^25^Hajdúchová *et al.* (2015)	Value-added tax impact on the state budget expenditures	Data from Customs and Tax administration	VAT calculation: VAT=price without tax × VAT rate VAT=price with tax/coefficient-	VAT can achieve state budget	Case study
9	^26^Guo (2021)	Impact of the VAT reduction policy on local fiscal pressure	-	-	VAT reduction important for development	Model based
10	^27^Wang *et al.* (2021)	Does tax deduction (VAT reform) relax financing constraints	Data from ASIF conducted by National Bureau of Statistics of China	Firm size, Firm age, Connections with government, Gross Domestic Product	VAT can achieve financial constraint	Experimental

Based on the findings from these 10 papers, the consumption tax, which is value-added tax, is important to the economy. Value-added tax is proved to bring positive impact and achieve the budget goal of financial constraint. Hence, the findings clearly indicate that VAT provides a stable revenue base, playing a key role as an incentive to control costs, and promote savings in IHL. Thus, these findings have proved that digital taxation is able to achieve the frugal innovation objective of this study.

## Discussion

In periods of economic slowdown, organisations encounter severe financial constraints. In this paper, a theoretical examination of digital taxation may be able to solve financial constraints and ensure IHL sustainability. In Industry 4.0, digital service tax policies are feasible tools for creating frugal innovation in IHL. To identify the core factors influencing digital taxation for frugal innovation, we examined relevant research papers and found that the core factors were gross domestic product growth, VAT calculation, GSDP and connections with the government. The results of previous studies proved the positive impact of digital taxation on the financial constraints encountered by organisations. We argue that VAT is essential for IHL. IHL will be able to leverage the concept frugal innovation by focusing on digital taxation to counter the financial constraints. As pointed out by this systematic review, there is gap in the study: though seemingly related, frugal innovation is not fully embraced. All articles discussed in this paper have highlighted the impact of VAT on the performance of a firm. This warrants more research on frugal innovation, using digital taxation as a tool to leverage on VAT benefits, particularly for businesses, in this case, IHL, to turbocharge their financial strength.

**Figure 4.**
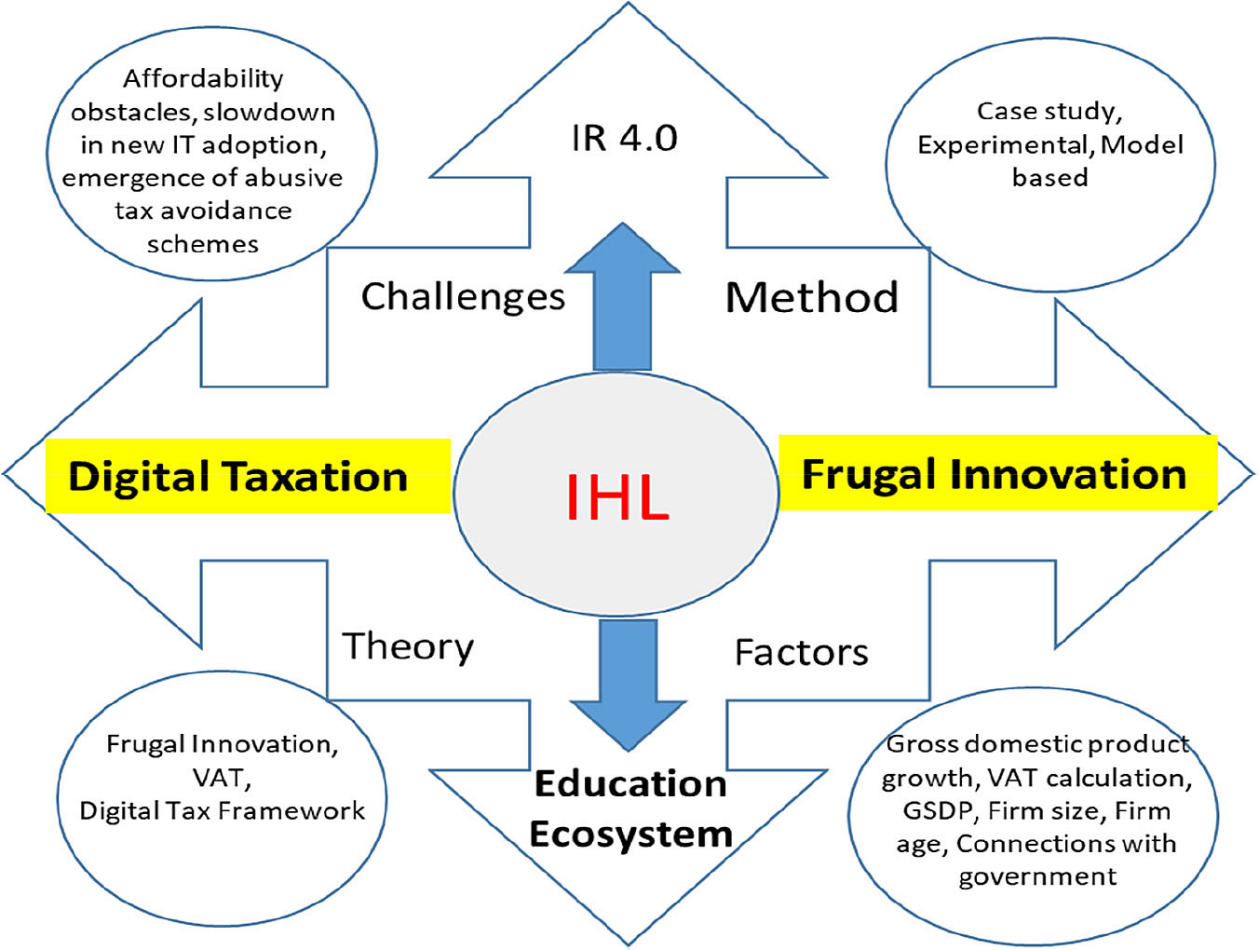
Proposed conceptual framework.

A limitation of this study is the number of keywords selected. Keyword selections are based on the research focus. However, it is possible to obtain more articles if the keywords are expanded to a field of study that is not specific in nature, such as municipality. This could possibly lead to publication biases ( Figure 4).

## Conclusion

The objective of this paper is to inform the digital taxation and frugal innovation community about the research gaps in studies on the contribution of digital taxation to frugal innovation published in the past two decades. We applied the online database methodology to published papers.. We adopted this methodology to determine the extent to which digital taxation is applied to promote frugal innovation. From 21 papers on digital taxation, the keyword search yielded 10 papers examining digital taxation for frugal innovation. From our review of the 10 papers, we provided two recommendations for future research. Firstly, for digital tax and VAT, the location of students taking a course should be considered, as billing and permanent addresses may vary. Secondly, research on the application of digital taxation to the actual concept of frugal innovation is limited. Thus, applied research in the future is necessary.

## Data availability

### Underlying data

All data underlying the results are available as part of the article and no additional source data are required.

### Extended data

Figshare: Data File – Digital Taxation.xls, https://doi.org/10.6084/m9.figshare.16722904.v2. ^28^

Data are available under the terms of the Creative Commons Attribution 4.0 International license (CC-BY 4.0).

### Reporting guidelines

PRISMA checklist for “Digital taxation to promote frugal innovation in institutions of higher learning: a three-decade systematic literature review”, https://doi.org/10.6084/m9.figshare.16722955.v1. ^29^

## References

[1] Che Omar R : Hubungan Di Antara Tanggapan Gaya Kepimpinan, Komunikasi Kepimpinan Dan Komitment Staff Pentadbiran Terhadap Kualiti Perkhidmatan IPT Swasta Di Malaysia (Doctoral Dissertation, Universiti Utara Malaysia). 2016.

[2] Ahmed S Masud MM : Measuring service quality of a higher educational institute towards students’ satisfaction. *Am. J. Educ. Res.* 2014;2(7):447–455. 10.12691/education-2-7-3

[3] Dahlan ARA Ibrahim J Razi M : *Redesign Business Model Options for — University of the Future ‖ and Staying Relevant in the Fourth Industrial Revolution Age, 2018.* Department of Information Systems Seminar;2018.

[4] Tiwari R Fischer L Kalogerakis K : Frugal innovation in Germany: A qualitative analysis of potential socio-economic impacts (No. 96). 2017.Working paper.

[5] Weyrauch T Herstatt C : What is frugal innovation? Three defining criteria. *J. Frugal Innova.* 2017;2(1):1. 10.1186/s40669-016-0005-y

[6] Pansera M : Frugal or fair? The unfulfilled promises of frugal innovation. *Technol. Innov. Manag. Rev.* 2018;8(4):6–13. 10.22215/timreview/1148

[7] Zeschky M Widenmayer B Gassmann O : Frugal innovation in emerging markets. *Res. Technol. Manag.* 2011;54(4):38–45. 10.5437/08956308X5404007

[8] Womack J Jones D Roos D : *The Machine That Changed the World.* New York, NY: Harper-Collins;1991.

[9] Hossain M Simula H Halme M : Can frugal go global? Diffusion patterns of frugal innovations. *Technol. Soc.* 2016;46:132–139. 10.1016/j.techsoc.2016.04.005

[10] Lei H Ha ATL Le PB : How ethical leadership cultivates radical and incremental innovation: the mediating role of tacit and explicit knowledge sharing. *J. Bus. Ind. Mark.* 2019;35(5):849–862. 10.1108/JBIM-05-2019-0180

[11] Sharma A Jha S : Innovation from emerging market firms: what happens when market ambitions meet technology challenges?. *J. Bus. Ind. Mark.* 2016;31(4):507–518. 10.1108/JBIM-12-2014-0265

[12] Bhattarai K Dung Thi KN Chan VN : Impacts of direct and indirect tax reforms in vietnam: A CGE analysis. *Economies.* 2019;7(2). 10.3390/economies7020050

[13] Pistone P Weber D : *Taxing the digital economy: The eu proposals and other insights.* ProQuest Ebook Central;2019. Reference Source

[14] Katz R : The impact of taxation on the digital economy 2015.Accessed 3 September 2020. Reference Source

[15] Alemu S : Meaning, Idea and History of University/Higher Education: Brief Literature Review. *FIRE: Forum for International Research in Education.* 2018;4. 10.32865/fire20184312

[16] Bunn D Assen E Enache C : *Digital taxation around the world.* Washingtion: Tax Foundation;2020. Reference Source

[17] Komba W : Increasing education access through open and distance learning in Tanzania: A critical review of approaches and practices. *Int. J. Educ. develop. using ICT.* 2009;5(5):8–21.

[18] Tranfield D Denyer D Smart P : Towards a methodology for developing evidence-informed management knowledge by means of systematic review. *Br. J. Manag.* 2003;14(3):207–222. 10.1111/1467-8551.00375

[19] Shahid HD Shaikh N Shah P : Pak-japan comparative study of consumption tax (value added tax) and its effects on economic growth rate and gross savings. *J. Economics and Political Economy.* 2019;6(3):295–303. 10.1453/jepe.v6i3.1938

[20] Hodzic S Celebi H : Value-added Tax and its Efficiency: EU–28 and Turkey. *UTMS J. Economics.* .2017;8(2):79–90. Reference Source

[21] Khan A Shadab N : Impact of value-added tax (VAT) revenue in major states of india. *Romanian Journal of Fiscal Policy.* 2013;4(1):27–46. Reference Source

[22] Kusumanto B : Incidence of value-added tax in indonesia: A general equilibrium analysis (Order No. 8924870). Available from ProQuest Dissertations & Theses Global. (303763470). 1989. Reference Source

[23] Koniagina MN : *IOP Conf. Ser.: Mater. Sci. Eng.* 2020;940:012040.

[24] Beebeejaun A : VAT on foreign digital services in Mauritius; a comparative study with South Africa. *Int. J. Law and Manag.* 2021;63(2):239–250. 10.1108/IJLMA-09-2020-0244

[25] Hajdúchová I Sedliačiková M Viszlai I : Value-added tax impact on the state budget expenditures and incomes. *Procedia Economics and Finance.* 2015;34:676–681. 10.1016/S2212-5671(15)01685-8

[26] Guo YM Shi YR : Impact of the VAT reduction policy on local fiscal pressure in China in light of the COVID-19 pandemic: A measurement based on a computable general equilibrium model. *Economic Analysis and Policy.* 2021;69:253–264. 10.1016/j.eap.2020.12.010 PMC9183237 35702722

[27] Wang J Shen G Tang D : Does tax deduction relax financing constraints? Evidence from China's value-added tax reform. *China Econ. Rev.* 2021;67:101619. 10.1016/j.chieco.2021.101619

[28] Dorasamy M Kwan WL : Data File.pptx. figshare. *Dataset.* 2021. 10.6084/m9.figshare.16722904.v2

[29] Dorasamy M Kwan WL : PRISMA checklist figshare. *Dataset.* 2021. 10.6084/m9.figshare.16722955

