# Supplementary material for: Digital taxation to promote frugal innovation in institutions of higher learning: a three-decade systematic literature review
Source: F1000Res. 2022 Mar 22;10:1055. Originally published 2021 Oct 18. [Version 2] doi: 10.12688/f1000research.73318.2 (PMC8984211; doi:10.12688/f1000research.73318.2)
Supplement: Peer review history

# Peer review: Reviewer response for version 2

**DOI:** 10.5256/f1000research.121964.r128305

**Published:** 2022-04-05

**Authors:** Muthaloo Subramaniam

**Affiliations:** 1 Faculty of Business, Management & Information Technology, Asia Metropolitan University, Johor, Malaysia

The authors have addressed all the proposed comments in the revised work.

Are the rationale for, and objectives of, the Systematic Review clearly stated?

Partly

Is the statistical analysis and its interpretation appropriate?

Not applicable

Are sufficient details of the methods and analysis provided to allow replication by others?

Yes

Are the conclusions drawn adequately supported by the results presented in the review?

Partly

Reviewer Expertise:

Taxation, Accounting and Information System, Marketing, Human Resource Management.

I confirm that I have read this submission and believe that I have an appropriate level of expertise to confirm that it is of an acceptable scientific standard.

# Peer review: Reviewer response for version 2

**DOI:** 10.5256/f1000research.121964.r128306

**Published:** 2022-03-31

**Authors:** Noor Adwa Sulaiman

**Affiliations:** 1 Faculty of Business and Economics, Universiti Malaya, Kuala Lumpur, Malaysia

The authors have addressed all of the comments.

Are the rationale for, and objectives of, the Systematic Review clearly stated?

Partly

Is the statistical analysis and its interpretation appropriate?

Yes

Are sufficient details of the methods and analysis provided to allow replication by others?

Yes

Are the conclusions drawn adequately supported by the results presented in the review?

Partly

Reviewer Expertise:

Auditing, corporate governance and financial reporting.

I confirm that I have read this submission and believe that I have an appropriate level of expertise to confirm that it is of an acceptable scientific standard.

# Peer review: Reviewer response for version 1

**DOI:** 10.5256/f1000research.76962.r118834

**Published:** 2022-02-07

**Authors:** Noor Adwa Sulaiman

**Affiliations:** 1 Faculty of Business and Economics, Universiti Malaya, Kuala Lumpur, Malaysia

This is an interesting paper to read. There are some areas that need further clarification. For example:

1. Concept of frugal innovation (FI) in IHL - in this case ODL or educational ecosystem is not clearly explained. Discussion about the conceptual link between FI and ODL or educational ecosystem is absent.  
2. Key findings (based on review of literature) on digital taxation (DT) within the context of IHL and ODL or educational ecosystem need to be emphasised - this is absent in the paper - as a result,  all of the research questions are not well answered.
3. The concept of educational ecosystem in the research questions and objectives is unclear. 
4. Discussion section -  it's a general discussion, this is not discussed within the context of the study - ODL or educational ecosystem.
5. Table 6 - none of the core papers are related to the objectives of the study - DT within IHL - this would then give effect to the findings of the study. 

Are the rationale for, and objectives of, the Systematic Review clearly stated?

Partly

Is the statistical analysis and its interpretation appropriate?

Yes

Are sufficient details of the methods and analysis provided to allow replication by others?

Yes

Are the conclusions drawn adequately supported by the results presented in the review?

Partly

Reviewer Expertise:

Accounting - auditing. Corporate governance and financial reporting.

I confirm that I have read this submission and believe that I have an appropriate level of expertise to confirm that it is of an acceptable scientific standard, however I have significant reservations, as outlined above.

# Response

**Published:** 2022-02-28

**Authors:** Magiswary Dorasamy

**Affiliations:** MULTIMEDIA UNIVERSITY, Malaysia

We thank you for your kind comments and feedback. We have compiled the suggestions and comments as per your call, and have addressed the concerns based on your excellent suggestion as follows:-

**Comment**: This is an interesting paper to read. There are some areas that need further clarification. For example:

**Response**: Thank you so much

**Comment**: Concept of frugal innovation (FI) in IHL - in this case ODL or educational ecosystem is not clearly explained. Discussion about the conceptual link between FI and ODL or educational ecosystem is absent. 

**Response**: Thank you for your suggestion. The linkages between FI and ODL is now rewritten as in the page 4 and 5, last paragraph. Educational ecosystem is now removed from this paper to stay focused on FI and Digital Tax in IHLs.

**Comment**: Key findings (based on review of literature) on digital taxation (DT) within the context of IHL and ODL or educational ecosystem need to be emphasised - this is absent in the paper - as a result,  all of the research questions are not well answered.

**Response**: The findings shows there is no article on DT within the context of IHL and ODL. Hence, we are unable to give more description on this context.

 Therefore, we looked into articles related to digital taxation and IHL only.

 Our findings are based both of the above. The result is shown in Table 3

**Comment**: The concept of educational ecosystem in the research questions and objectives is unclear.

**Response**: Thank you for your comment. We have now removed the educational eco-system concept as this paper focuses only on digital taxation in IHLs.

**Comment**: Discussion section -  it's a general discussion, this is not discussed within the context of the study - ODL or educational ecosystem.

**Response**: Thank you for your comment. The discussion has been revised as suggested in page 11 under Discussion heading.

 The Education ecosystem concept is now removed.

**Comment**: Table 6 - none of the core papers are related to the objectives of the study - DT within IHL - this would then give effect to the findings of the study.

**Response**: Based on Table 3 Summary of keyword search, we have stated that there is zero paper related to Digital Taxation and IHL. Hence, only papers related to digital taxation is given.

 Since no papers on DT in IHL, we argue that there is a need to fill this research gap and this warrants for more research.

**Overall**: The paper has been repositioned to show the more rigor and relevance as per the review comments. The content has been strengthened with detail discussion. We trust that the paper reads much better now and that we have addressed all major concerns.

 Thank you.

# Peer review: Reviewer response for version 1

**DOI:** 10.5256/f1000research.76962.r119302

**Published:** 2022-02-03

**Authors:** Muthaloo Subramaniam

**Affiliations:** 1 Faculty of Business, Management & Information Technology, Asia Metropolitan University, Johor, Malaysia

This is indeed a great topic to be researched. Nevertheless, it can be enhanced accordingly. Thus, it is worth sharing some comments for the submitted article as follow:

**Major points:**

- Introduction, "IHL are seen as crucial...": Ensure that private IHLs are facing similar issues with those public IHLs, or else it cannot be generalized to entire IHL whilst focus is on all the IHLs in Malaysia.
- Frugal Innovation, "The philosophy of frugal innovation..." This is rather vague and how can this be related to Malaysia? Originated from emerging and developed economies - but reflects onto Malaysia? Not clearly stated. This part can be improved.
- Frugal Innovation, "Besides, frugal innovation consists...": Provide examples on environmental benefits and business opportunities.
- Frugal Innovation, "Although fugal innovation lacks...": 
  - Spelling error. Should be "frugal"?
  - How to provide the high-quality services without using high technology? Needs discussion.
- Digital Taxation, "Currently, Malaysia has 20 public universities...": Not organized appropriately. The discussion is not well connected and concluded here. What is different between federal government and public sector - where can funds for public sector be expected? 

**Other points:**

- Use IHLs instead of IHL.
- "... *would be* implemented in January 2020." Ensure the use of proper tense.
- Digital tax exemption is allowed for ODL - what is next? This is rather incomplete. 
- VAT, GST or SST? Ensure consistency with what Malaysia has implemented as to date. Perhaps, the VAT related to the country that it was implemented can be retained. 
- Digital taxation has been implemented only on 1st January 2020. How are the reviews done in other countries similar to such tax implemented here in Malaysia? How relevant, that leads to implementation (if any)? What would be the post implementation benefits to the country? Suggested to discuss.  
- Be consistent with the term "ecosystem" or "educational ecosystem". These terms appeared in research questions without being adequately explained before using them. 
- Ensure the first letter is capitalized when using term such as -  gross state domestic product. It would be appropriate for such words to be mentioned such as Gross State Domestic Product (GSDP).
- The scope of digital taxation was not clearly explained - to what extent does it overlap with, and what is the relationship with, consumption tax - as discussed both consumption tax and digital services tax cover digital goods and services (refer to Table 1). Suggested to clarify.
- Discuss the relationship between the frugal innovation and ODL in IHLs. Would you think exempted ODL for tax purposes would be beneficial for the country in terms of revenue generation? 
- Suggested to use active English and avoid using the word "we" knowing who were the authors of this work. 
- Can the Covid19 pandemic pose any challenges since the implementation of digital tax on the 1st January 2020? Suggested to discuss.

Are the rationale for, and objectives of, the Systematic Review clearly stated?

Partly

Is the statistical analysis and its interpretation appropriate?

Not applicable

Are sufficient details of the methods and analysis provided to allow replication by others?

Yes

Are the conclusions drawn adequately supported by the results presented in the review?

Partly

Reviewer Expertise:

Taxation, Accounting and Information System, Marketing, Human Resource Management.

I confirm that I have read this submission and believe that I have an appropriate level of expertise to confirm that it is of an acceptable scientific standard, however I have significant reservations, as outlined above.

# Response

**Published:** 2022-02-28

**Authors:** Magiswary Dorasamy

**Affiliations:** MULTIMEDIA UNIVERSITY, Malaysia

We thank you for your valuable comments and suggestions. We have compiled the suggestions and comments as per your call, and have addressed the concerns based on your excellent suggestion as follows:-

**Comment**: This is indeed a great topic to be researched. Nevertheless, it can be enhanced accordingly. Thus, it is worth sharing some comments for the submitted article as follow:

**Response**: Thank you for your comment.

**Comment**: Introduction, "IHL are seen as crucial...": Ensure that private IHLs are facing similar issues with those public IHLs, or else it cannot be generalized to entire IHL whilst focus is on all the IHLs in Malaysia.

**Response**: Thank you for the suggestion. We have now included both private and public IHLs in Malaysia. It has been revised as suggested in page 2.

**Comment**: Frugal Innovation, "The philosophy of frugal innovation..." This is rather vague and how can this be related to Malaysia? Originated from emerging and developed economies - but reflects onto Malaysia? Not clearly stated. This part can be improved.

**Response**: We have now rewrote (page 3 paragraph2) the sentence to the following: “The philosophy of frugal innovation is considered as an innovative solution originated in developing countries and has become a concern for people in developed economies.”

**Comment**: Frugal Innovation, "Besides, frugal innovation consists...": Provide examples on environmental benefits and business opportunities.

**Response**: We have now rewrote (page 3 paragraph 2) the sentence to the following: “With the right conditions, frugal innovation can provide environmental benefit in term of improvement in the lives of people facing live constraints. In terms of business opportunities, frugal innovation refers to efforts by businesses to improve their products and services through a low-cost process.”

**Comment**: Frugal Innovation, "Although fugal innovation lacks...":

- Spelling error. Should be "frugal"?
- How to provide the high-quality services without using high technology? Needs discussion.

**Response**: We have corrected the spelling error.

 Added the following line to describe the sentence on high quality service:

 “Instead of consuming scarce resources, business can use frugal innovation to develop and sell more sustainable products and services.”

**Comment**: Digital Taxation, "Currently, Malaysia has 20 public universities...": Not organized appropriately. The discussion is not well connected and concluded here. What is different between federal government and public sector - where can funds for public sector be expected?

**Response**: Thank you for the comment. We have removed this paragraph.

**Comment**: Use IHLs instead of IHL.

**Response**: It has been revised as suggested.

**Comment**: "...would be implemented in January 2020." Ensure the use of proper tense.

**Response**: It has been revised as follows: “Digital tax was implemented in January 2020, as indicated in 2019 Malaysian national budget.”

**Comment**: Digital tax exemption is allowed for ODL - what is next? This is rather incomplete.

**Response**: It has been revised as suggested. The following text is now added: “Since the education sectors are in high demand in delivering education through online teaching, thus tax concerns, such as value-added taxes (VAT) are given high emphasis. This is because the ODL courses will be exposed to tax and the tax regulations, and influence the viability of the course offered.”

**Comment**: VAT, GST or SST? Ensure consistency with what Malaysia has implemented as to date. Perhaps, the VAT related to the country that it was implemented can be retained.

**Response**: This paper focuses on digital tax which is VAT for ODL only. GST and SST are not applicable in this paper.

**Comment**: Digital taxation has been implemented only on 1st January 2020. How are the reviews done in other countries similar to such tax implemented here in Malaysia?

 How relevant, that leads to implementation (if any)?

 What would be the post implementation benefits to the country? Suggested to discuss. 

**Response**: As digital tax is new in Malaysia, we provide the examples of digital tax in other countries. It has been revised as suggested.

**Comment**: Be consistent with the term "ecosystem" or "educational ecosystem". These terms appeared in research questions without being adequately explained before using them.

**Response**: It has been revised as suggested. The term educational ecosystem is now removed from this paper.

**Comment**: Ensure the first letter is capitalized when using term such as -  gross state domestic product. It would be appropriate for such words to be mentioned such as Gross State Domestic Product (GSDP).

**Response**: It has been revised as suggested.

**Comment**: The scope of digital taxation was not clearly explained - to what extent does it overlap with, and what is the relationship with, consumption tax - as discussed both consumption tax and digital services tax cover digital goods and services (refer to Table 1). Suggested to clarify.

**Response**: Consumption tax is a type of digital tax, as per categorize in table 1.  They don’t overlap.

**Comment**: Discuss the relationship between the frugal innovation and ODL in IHLs.

 Would you think exempted ODL for tax purposes would be beneficial for the country in terms of revenue generation?

**Response**: We have now added more discussion on the relationship frugal innovation and ODL in IHLs (page 4, last paragraph).

 At present, digital tax for ODL is exempted. It is definitely a lost of revenue for IHLs as well as country. In this paper, we argue that the digital tax should be extended to ODL, and this will be a frugal innovation solution for IHLs. 

**Comment**: Suggested to use active English and avoid using the word "we" knowing who were the authors of this work.

**Response**: Thank you for your comment. It has been revised as suggested.

**Comment**: Can the Covid19 pandemic pose any challenges since the implementation of digital tax on the 1st January 2020? Suggested to discuss.

**Response**: Thank you for your comment. It has been revised as suggested. Please see page 4.  The following sentence is now added to indicate the impact of Covid19: “In particular, during Covid19 pandemic, the adoption of online teaching method has become a necessity for IHLs to continue its teaching and learning process.”

**Overall:** The paper has been repositioned to show the more rigor and relevance as per the review comments. The content has been strengthened with detail discussion. We trust that the paper reads much better now and that we have addressed all major concerns.

 Thank you.
